# The Suzuki Reaction Applied to the Synthesis of Novel Pyrrolyl and Thiophenyl Indazoles 

**DOI:** 10.3390/molecules17044508

**Published:** 2012-04-16

**Authors:** Antonella Migliorini, Chiara Oliviero, Tecla Gasperi, Maria Antonietta Loreto

**Affiliations:** 1 Department of Chemistry, “Sapienza” University of Rome, P.le A. Moro 5, I-00185 Roma, Italy; Email: antonella.migliorini@gmail.com (A.M.); chiara.oliviero@yahoo.it (C.O.); 2 Department of Mechanical and Industrial Engineering and CISDiC, University of Studies “Roma Tre”, via della Vasca Navale 79, I-00146 Roma, Italy; Email: tgasperi@uniroma3.it

**Keywords:** indazoles, pyrrole, thiophene, Suzuki cross-coupling, heterobiaryl compounds

## Abstract

The paper describes the Suzuki cross-coupling of a variety of *N *and *C*-3 substituted 5-bromoindazoles with *N*-Boc-2-pyrrole and 2-thiopheneboronic acids. The reactions, performed in the presence of K_2_CO_3_, dimethoxyethane and Pd(dppf)Cl_2_ as catalyst, gave the corresponding adducts in good yields. The methodology allows the facile production of indazole-based heteroaryl compounds, a unique architectural motif that is ubiquitous in biologically active molecules.

## 1. Introduction

**Figure 1:**
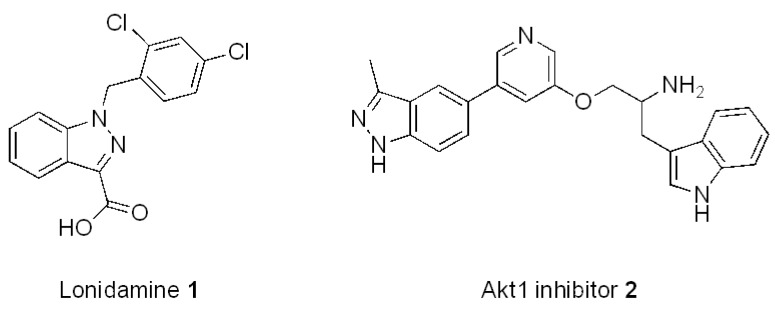
Relevant molecules with an indazole moiety.

Indazole, the indole bioisoster, is a highly utilized pharmacophore [1] found in many biologically active compounds such as lonidamine (**1**) [2], a molecule with anticancer activity, or the Akt1 inhibitor **2** (Figure 1) [3].

Due to the broad variety of their biological activities, the synthesis of indazole derivatives as well as the functionalization of the indazole ring system have recently been reviewed [4,5,6,7,8,9,10,11,12], especially in the context of drug development. During the last years, indazole derivatives bearing aryl groups on the 5 or 6 position have been prepared and identified as potent, selective glucocorticoid receptor agonists and antagonists [13] or inhibitors of protein kinase c-zeta [14]. Conversely, to the best of our knowledge, the functionalization of the indazole ring with aromatic heterocycles like pyrrole and thiophene has been less explored. Among the very few reported examples, some recent patents have described 3-substituted-5-thienyl-1*H-*indazole as ligands for nicotinic acetylcholine receptors [15] or inhibitors of kinase activity [16,17]. Likewise, only 6-pyrrolyl-indazoles have recently been disclosed for their inhibitory activity of glycogen synthase kinase-3, and their synthesis was performed starting from pyrrolylbenzonitriles [18].

As part of the effort to discover novel indazole derivatives as valuable building blocks in medicinal chemistry [19], we were looking for an efficient and effective synthetic protocol of wide applicability towards 5-(pyrrol-2-yl)- and 5-(thiophen-2-yl)-1*H*-indazoles. The Suzuki reaction provides a very reliable method for the preparation of biaryl derivatives [20]. However, although simple aryl halides and aryl boronic acids are widespread employed coupling partners, the corresponding reactions involving their heteroaryl analogues are noticeably fewer [21,22,23,24,25,26,27,28]. Herein, we report our initial investigations on the Suzuki cross-coupling between differently *N-*substituted 5-bromo-indazoles and pyrrole- or thiopheneboronic acids.

## 2. Results and Discussion

**Scheme 1:**
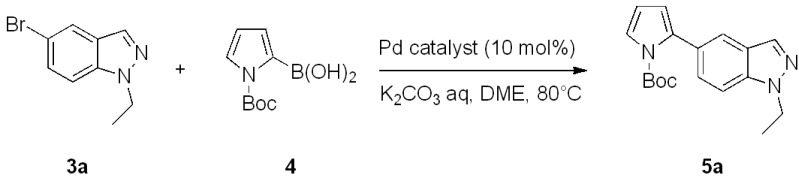
Suzuki cross-coupling of 5-bromo-1-ethyl-1*H*-indazole and *N*-Boc-2-pyrroleboronic acid.

In order to determine the optimal reaction conditions we began by studying the cross-coupling of 5-bromo-1-ethyl-1*H*-indazole (**3a**) with *N*-Boc-2-pyrroleboronic acid (**4**) [29] as a pilot reaction (Scheme 1). Indazole **3a** was prepared by the alkylation of the 5-bromo-1*H*-indazole with ethyl bromide [30]. In the presence of cesium carbonate (Cs_2_CO_3_), a 1.2:1 ratio of **3a** and the *N*-2 isomer **3g** was obtained. The two regioisomers were purified and identified by comparison of their spectral data with that reported for similar *N*-alkylated indazoles [31].

The Suzuki reaction was carried out by employing K_2_CO_3_ as base, dimethoxyethane as solvent and heating the reaction mixture at 80 °C. As shown in Table 1, we examined four palladium catalysts and found that [1,1'-bis(diphenylphosphino)ferrocene]palladium(II) dichloride [Pd(dppf)Cl_2_] [32] was the best choice, affording the coupling product in high yield after only two hours. Interestingly, bis(tricyclohexylphosphine)palladium [Pd(PCy_3_)_2_] yielded the product in modest yield, although generally the electron richness and the sterical hindrance of the phosphinic ligands make it an efficient palladium source for cross-coupling reaction [33,34]. The commonly used tetrakis(triphenyl-phosphine)palladium [Pd(PPh_3_)_4_] and bis(triphenylphosphine)palladium(II) dichloride [Pd(PPh_3_)2Cl_2_] were less effective than [Pd(dppf)Cl_2_] for this transformation, affording the final product after longer reaction times and in lower yields.

**Table 1:** Screening of palladium catalysts for the Suzuki coupling of 5-bromo-1-ethyl-1*H*-indazole and *N*-Boc-2-pyrroleboronic acid.

Entry	Pd catalyst	Reaction Time	5a Yield
1	Pd(PPh_3_)_4_	4 h	22%
2	Pd(PPh_3_)_2_Cl_2_	4 h	75%
3	Pd(PCy_3_)_2_	2 h	57%
4	Pd(dppf)Cl_2_	2 h	84%

**Scheme 2:**
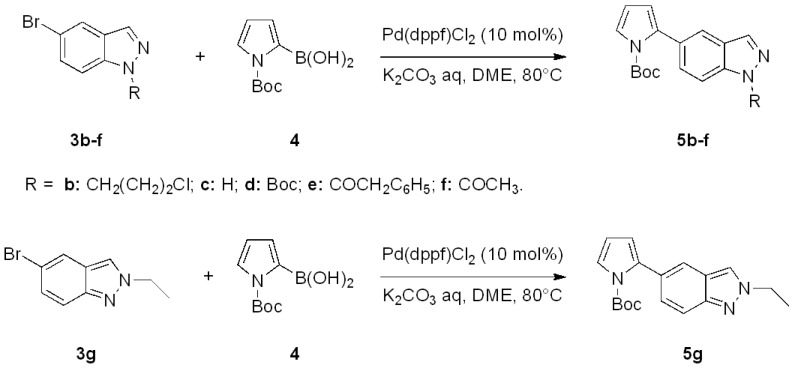
Synthesis of 5-(pyrrol-2-yl)-1*H*-indazoles by the Suzuki cross-coupling.

Having identified Pd(dppf)Cl_2_ as the most suitable catalyst, in order to explore the versatility of this type of Suzuki coupling, a series of 5-bromoindazoles bearing alkyl or acyl groups on the *N*-1 or *N*-2 positions were prepared [30,35,36,37,38] and tested with Boc-protected-2-pyrroleboronic acid **4** (Scheme 2).

In all cases the expected coupling products were obtained in very modest to quite good yields and fully characterized (Table 2). The lower yields registered for the *N*-acyl-indazoles **3e** and **3f** may be a consequence of the facile deacylation of these substrates under basic conditions [39]. This is confirmed by the isolation of **5c** (30% yield) as an additional product in their reaction mixtures. The reaction was also performed on the unsubstituted 5-bromoindazole **3c** and afforded the corresponding product **5c** in 50% yield, due to the likely formation of side-products, not further investigated. Moreover, it is worthy to note that the *N*-Boc-indazole **3d** resulted to be a very good substrate for the cross-coupling. The easy removal of the Boc group would make the coupling product **5d** a valuable building block in the synthesis of new interesting indazole-based molecules.

On the basis of these positive results, we extended the scope of the Suzuki cross-coupling to the synthesis of 5-(thiophen-2-yl)-1*H*-indazoles. Thiophene, like pyrrole, is found in a variety of natural products and pharmaceutically interesting compounds [40]. In addition, polythiophenes, which are often prepared via Suzuki-Miyaura processes, are highly conducting polymers that possess good processing qualities [41].

**Scheme 3:**
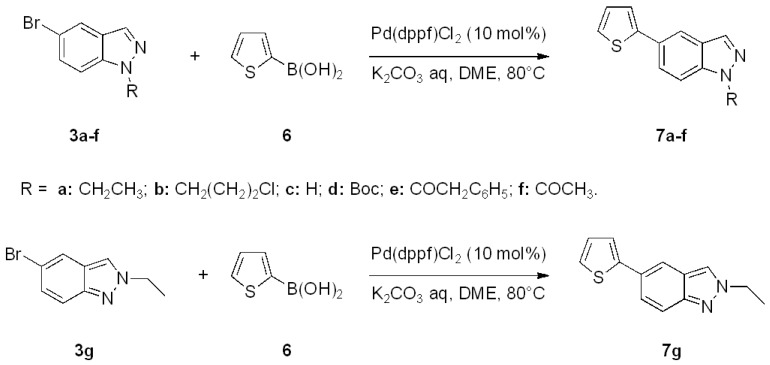
Synthesis of 5-(thiophen-2-yl)-1*H*-indazoles by the Suzuki cross-coupling.

The coupling with 2-thiopheneboronic acid (**6**) was carried out under the same reaction conditions previously employed and gave the expected 5-(thiophen-2-yl)-1*H*-indazoles **7a−g** (Scheme 3).

With respect to the corresponding coupling reactions with 2-pyrroleboronic acid **4**, the products were obtained in lower yields (Table 2), due to the tendency of thiopheneboronic acids to undergo protodeboronation and the formation of a side-product identified as the thiophene dimer [22].

**Table 2:** Suziki cross-coupling reaction for the synthesis of 5-(pyrrol-2-yl)- and 5-(thiophen-2-yl)-1*H*-indazoles.

Entry	Products 5	Yield 5 ^[a]^	Products 7	Yield 7 ^[a]^
a	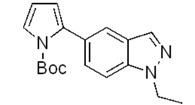	84%	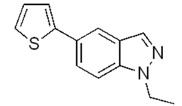	60%
b	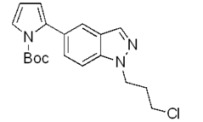	74%	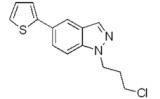	62%
c	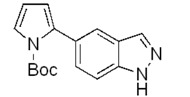	50%	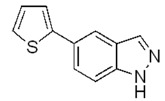	traces
d	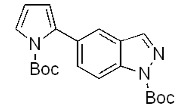	81%	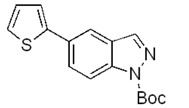	70%
e	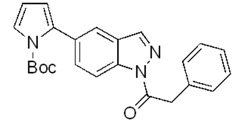	45%	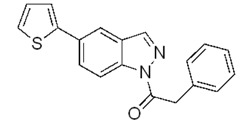	30%
f	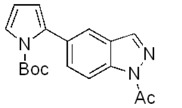	30%	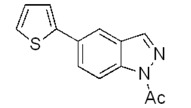	35%
g	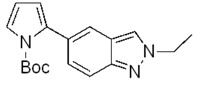	92%	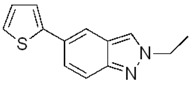	87%

^[a]^ Isolated Yields.

**Scheme 4:**
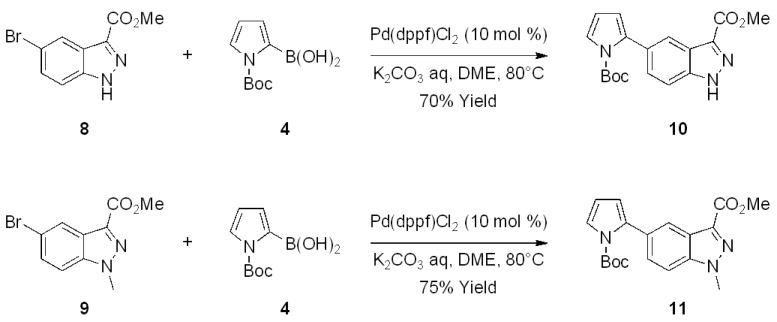
Synthesis of 3-substituted-(5-pyrrol-2-yl)-indazoles by the Suzuki cross-coupling.

On the bases of the described successful results and in view of the interest in *C*-3 substituted indazole derivatives reported in the literature [5], a preliminary study for the extension of the above reaction to *C*-3 substituted indazoles has been also initiated. To this purpose, 5-bromo-1*H*-indazole-3-carboxylic acid methyl ester (**8**, Scheme 4) was prepared by a known esterification of 5-bromo-1*H*-indazole-3-carboxylic acid, reported to afford **8** as the unique product [42]. However, in our hands, the protocol gave a 1:1 mixture of two products identified as **8** and the corresponding unprecedented 1-methyl derivative **9**, respectively.

Therefore, both substrates **8** and **9** were reacted with the Boc-protected-2-pyrroleboronic acid **4** (Scheme 4) and gave the corresponding 3-substituted-(5-pyrrol-2-yl)-indazoles **10** and **11**, thus indicating that the *C*-3 substituent doesn’t invalidate the success of the Suzuki reaction. The extension of this methodology to variously *C*-3 functionalized indazole derivatives by using pyrrole and thiophene boronic acids is currently under investigation. 

## 3. Experimental

### 3.1. General Experimental Methods

Solvents and common reagents were purchased from a commercial source and used without further purification. *N*-Boc-2-pyrroleboronic acid **4** was prepared according to the literature procedure [29]. 2-Thiopheneboronic acid **6** and 5-bromo-1*H*-indazole-3-carboxylic acid were purchased from a commercial source. All reactions were monitored by thin layer chromatography (TLC) carried out on Merck F-254 a silica glass plates and visualized with UV light. The resulting mixtures were purified by flash column chromatography on silica gel by eluting, unless otherwise stated, with hexane/ethyl acetate, 8:2. ^1^H-NMR spectra were recorded on Varian Gemini 300 (300 MHz) instrument. Chemical shifts are expressed in parts per million (δ scale) and are referenced to the residual protons of the NMR solvent (CHCl_3_: δ 7.26); (s) = singlet; (d) = doublet; (t) = triplet; (q) = quartet; (dd) = double doublet; (ddd) = double double doublet; (dt) = double triplet; (dq) = double quartet; (m) = multiplet. Coupling constants (*J*) are expressed in Hz. ^13^C-NMR spectra were recorded on Varian Gemini 300 (75 MHz). Chemical shifts are expressed in parts per million (δ scale) and are referenced to the residual carbons of the NMR solvent (CHCl_3_: δ 77.0). Infrared Spectra (IR) were obtained using a Perkin-Elmer 1600 (FT-IR, Walthman, MA, USA); data are presented as the frequency of absorption (cm^−1^). HRMS Spectra were recorded with Micromass Q-TOF *micro* Mass Spectrometer (Waters, Milford, MA, USA).

*5-Bromo-1-ethyl-1H-indazole* **3a** [30]. To a solution of 5-bromo-1*H*-indazole (500 mg, 2.55 mmol) in anhydrous DMF (8 mL), ethyl bromide (3.85 mmol, 0.60 mL) was added at room temperature, followed by an excess of Cs_2_CO_3_ (2.5 g, 7.65 mmol). After stirring the reaction mixture at room temperature for 3 h, 2 N HCl was added until a neutral pH was reached. The aqueous layer was extracted with ethyl acetate and the combined organic extracts were dried over anhydrous Na_2_SO_4_. After filtration, the solvent was removed in *vacuo *and the residue was purified by flash column chromatography. Yield: 227 mg (1 mmol, 40%); dark orange viscous liquid; *R_f_* = 0.47. ^1^H-NMR: *δ* = 1.45 (t, 3H, CH_3,_ *J* = 7.3 Hz), 4.35 (q, 2H, CH_2_, *J* = 7.3 Hz), 7.21 (d, 1H, ArH, *J* = 8.9 Hz), 7.36 (dd, 1H, ArH, *J* = 1.8 Hz, *J *= 8.9 Hz), 7.77–7.81 (m, 1H, ArH), 7.88 (s, 1H, ArH) ppm. ^13^C-NMR: *δ* = 15.1, 44.1, 110.5, 113.7, 123.7, 125.8, 129.3, 132.2, 137.9 ppm. HRMS: calcd. for C_9_H_10_BrN_2_ 225.0027; found 225.0032.

*5-Bromo-2-ethyl-2H-indazole* (**3g**) [30,43]. This compound was obtained in the alkylation of 5-bromo-1*H*-indazole with ethyl bromide together with **3a** (**3a**/**3g** = 1.2/1). Yield: 178 mg (0.80 mmol, 31%); dark orange viscous liquid; *R_f_* = 0.12. ^1^H-NMR: *δ* = 1.62 (t, 3H, CH_3_, *J* = 7.3 Hz), 4.45 (q, 2H, CH_2_, *J* = 7.3 Hz), 7.32 (dd, 1H, ArH, *J* = 1.8 Hz, *J* = 9.1 Hz), 7.58 (dd, 1H, ArH, *J* = 0.8 Hz, *J* = 9.1 Hz), 7.77−7.80 (m, 1H, ArH), 7.85 (s, 1H, ArH) ppm. ^13^C-NMR: *δ* = 15.9, 48.9, 111.4, 115.2, 119.3, 121.6, 122.4, 129.6, 147.4 ppm. HRMS: calcd. for C_9_H_10_BrN_2_ 225.0027; found 225.0027.

*5-Bromo-1-(3-chloro-propyl)-1H-indazole* (**3b**) [44]. Compound **3b** was prepared from 5-bromo-1*H*-indazole (500 mg, 2.55 mmol) and 1-bromo-3-chloropropane (3.85 mmol, 0.40 mL) according to the procedure described for **3a**. After solvent evaporation, the crude mixture was chromatographed over silica gel. Yield: 352 mg (1.3 mmol, 50%); dark orange viscous liquid; *R_f_* = 0.62. ^1^H-NMR: *δ* = 2.35–2.43 (m, 2H, CH_2_C*H*_2_CH_2_), 3.46 (t, 2H, C*H*_2_Cl, *J* = 5.8 Hz), 4.54 (t, 2H, CH_2_N, *J* = 6.0 Hz), 7.38 (dd, 1H, ArH, *J* = 0.8 Hz, *J* = 8.9 Hz), 7.46 (dd, 1H, ArH, *J* = 1.8 Hz, *J* = 8.9 Hz), 7.84−7.88 (m, 1H, ArH), 7.95 (s, 1H, ArH) ppm. ^13^C-NMR: *δ* = 32.6, 42.0, 45.7, 110.6, 113.9, 123.7, 125.6, 129.7, 132.9, 138.8 ppm. HRMS: calcd. for C_10_H_11_BrClN_2_ 272.9794; found 272.9800. The *N*-2 isomer was isolated as minor product. Yield: 69 mg (0.25 mmol, 10%); dark orange viscous liquid; *R*_f_ = 0.47. ^1^H-NMR: *δ* = 2.42−2.50 (m, 2H, CH_2_C*H*_2_CH_2_), 3.42 (t, 2H, C*H*_2_Cl, *J* = 5.8 Hz), 4.59 (t, 2H, CH_2_N, *J* = 6.4 Hz), 7.34 (dd, 1H, ArH, *J* = 1.8 Hz, *J* = 9.1Hz), 7.57 (dd, 1H, ArH, *J* = 0.8 Hz, *J* = 9.1 Hz), 7.79−7.83 (m, 1H, ArH), 7.92 (s, 1H, ArH) ppm. ^13^C-NMR: *δ* = 32.9, 41.6, 50.6, 110.3, 115.4, 119.4, 122.5, 123.3, 129.9, 147.8 ppm. HRMS: calcd. for C_10_H_11_BrClN_2_ 272.9794; found 272.9792. 

*5-Bromo-indazole-1-carboxylic acid tert-butyl ester* (**3d**). Compound **3d** was prepared from 5-bromo-1*H*-indazole (500 mg, 2.55 mmol) and Boc_2_O (583 mg, 2.68 mmol) according to the literature procedure and the spectral data were in agreement with those reported in the literature [36]. Yield: 558 mg (1.89 mmol, 74%).

*1-(5-Bromo-indazol-1-yl)-2-phenyl-ethanone* (**3e**). Compound **3e** was prepared from 5-bromo-1*H*-indazole (500 mg, 2.55 mmol) and phenylacetyl chloride (3.85 mmol, 0.50 mL) according to the procedure described for **3a**. After solvent evaporation, the crude mixture was chromatographed over silica gel. Yield: 360 mg (1.15 mmol, 45%); dark orange viscous liquid; *R*_f_ = 0.85. ^1^H-NMR: *δ* = 4.52 (s, 2H, CH_2_C=O), 7.25−7.50 (m, 5H, ArH), 7.63 (dd, 1H, ArH, *J* = 1.8, *J* = 8.8 Hz), 7.86−7.89 (m, 1H, ArH), 8.11 (s, 1H, ArH), 8.32 (dd, 1H, ArH, *J *= 0.7 Hz, *J* = 8.8 Hz) ppm. ^13^C-NMR: *δ* = 41.7, 117.1, 117.9, 120.3, 123.8, 127.5, 128.2, 128.9, 129.9, 132.7, 133.9, 139.0, 171.6 ppm. IR (CHCl_3_): 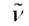 = 1728 cm^−1^. HRMS: calcd. for C_15_H_11_BrN_2_NaO 336.9952; found 336.9949.

*1-(5-Bromo-indazol-1-yl)-ethanone* (**3f**) [38]. To a solution of 5-bromo-1*H*-indazole (500 mg, 2.55 mmol) in anhydrous DCM (48 mL) was added acetic anhydride (0.45 mL, 5.10 mmol), pyridine (403 mg, 0.40 mL, 5.10 mmol) and dimethylaminopyridine (DMAP) in a catalytic amount. The solution was stirred at 40 °C overnight. The organic phase was washed with water (2 × 50 mL), 1N HCl (2 × 50 mL), NaHCO_3_ (aq) (2 × 50 mL) and brine (2 × 50 mL) and then dried over anhydrous Na_2_SO_4_. The solvent was removed *in vacuo* to give **3f**. Yield: 833 mg (3.50 mmol, 70%); dark orange viscous liquid; *R_f_* = 0.87. ^1^H-NMR: *δ* = 2.81 (s, 3H, CH_3_), 7.65 (dd, 1H, ArH, *J* = 1.8, *J* = 8.8 Hz), 7.87−7.90 (m, 1H, ArH), 8.08 (s, 1H, ArH), 8.34 (dd, 1H, ArH, *J* = 0.7 Hz, *J* = 8.8 Hz) ppm. ^13^C-NMR: *δ* = 22.9, 115.8, 116.6, 122.4, 126.8, 131.4, 136.7, 137.5, 169.9. IR (CHCl_3_): 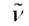 = 1726 cm^−1^. HRMS: calcd. for C_9_H_7_BrN_2_NaO 260.9639; found 260.9644.

*5-Bromo-1H-indazole-3-carboxylic acid methyl ester* (**8**) and *5-Bromo-1-methyl-1H-indazole-3-carboxylic acid methyl ester* (**9**). Compounds **8** and **9** were obtained from 5-bromo-1*H*-indazole-3-carboxylic acid (160 mg, 0.66 mmol) by applying an esterification procedure reported to afford **8** as the unique product [42]. However, in our hands, the protocol gave a 1:1 mixture of two products that were separated by chromatography. The first compound was identified as **8** and showed spectral data in agreement with those reported in literature [42]. Yield: 61 mg (0.24 mmol, 36%). The second compound was identified as **9**. Yield: 59 mg (0.22 mmol, 34%); *R*_f_ = 0.7 (hexane/ethyl acetate, 1:1). ^1^H-NMR: *δ* = 4.03 (s, 3H, CO_2_CH_3_), 4.48 (s, 3H, NCH_3_), 7.41 (dd, 1H, ArH, *J* = 1.8 Hz, *J* = 9.1 Hz), 7.63 (dd, 1H, ArH, *J* = 0.7 Hz, *J* = 9.1 Hz), 8.13−8.15 (m, 1H, ArH) ppm. ^13^C-NMR: *δ* = 41.8, 52.3, 119.3, 119.9, 123.7, 127.3, 128.9, 131.4, 145.9, 161.0 ppm. HRMS: calcd. for C_10_H_10_BrN_2_O_2_ 268.9926; found 268.9930.

*3.2. General Procedure for the Suzuki Coupling Reaction*

A solution of bromo indazole **3** (1 mmol) and [1,1'-bis(diphenylphosphino)ferrocene]palladium(II) dichloride [Pd(dppf)Cl_2_] (10%) in anhydrous DME (10 mL) was stirred under a flow of argon for 1 h. To the solution were added sequentially 1-(*tert*-butoxycarbonyl)pyrrole-2-boronic acid (**4**) or 2-thiopheneboronic acid (**6**) (2 mmol) in anhydrous DME (2.6 mL) and potassium carbonate (2 mmol) in water (2.5 mL). The mixture was heated to 80 °C for 2 h and allowed to cool. The reaction mixture was then poured into aqueous saturated NaHCO_3_ solution and extracted with ethyl acetate. The organic layers were combined, washed with brine and dried over Na_2_SO_4_. The solution was concentrated *in vacuo* and the residue was purified by flash column chromatography on silica gel to give the desired product.

*2-(1-Ethyl-1H-indazol-5-yl)-pyrrole-1-carboxylic acid tert-butyl ester *(**5a**). Yield: 261 mg (0.84 mmol, 84%); dark orange viscous liquid; *R*_f_ = 0.28. ^1^H-NMR: *δ* = 1.34 (s, 9H, CH_3_), 1.52 (t, 3H, CH_3_, *J* = 7.3 Hz), 4.44 (q, 2H, CH_2_, *J *= 7.3 Hz), 6.19−6.25 (m, 2H, ArH), 7.25−7.26 (m, 1H, ArH), 7.34−7.36 (m, 1H, ArH), 7.38 (s, 1H, ArH), 7.69 (m, 1H, ArH), 7.99 (s, 1H, ArH) ppm. ^13^C-NMR *δ* = 15.2, 27.9, 44.3, 83.7, 108.2, 110.5, 114.4, 121.2, 122.4, 123.7, 127.3, 129.1, 133.8, 135.2, 139.1, 149.6 ppm. IR (CHCl_3_): 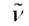 = 1733 cm^−1^. HRMS: calcd. for C_18_H_21_N_3_NaO_2_ 334.1531; found 334.1526.

*2-(2-Ethyl-2H-indazol-5-yl)-pyrrole-1-carboxylic acid tert-butyl ester* (**5g**). Yield: 286 mg (0.92 mmol, 92%); dark orange viscous liquid; *R*_f_ = 0.32 (hexane/ethyl acetate, 7:3). ^1^H-NMR: *δ* = 1.33 (s, 9H, CH_3_), 1.61 (t, 3H, CH_3,_*J* = 7.3 Hz), 4.46 (q, 2H, CH_2_, *J* = 7.3 Hz), 6.18−6.23 (m, 2H, ArH), 7.25 (dd, 1H, ArH, *J* =1.8 Hz, *J* = 8.9 Hz), 7.35−7.36 (m, 1H, ArH), 7.60 (m, 1H, ArH), 7.65 (dd, 1H, ArH, *J *= 0.8 Hz, *J *= 8.9 Hz), 7.99 (s, 1H, ArH) ppm. ^13^C-NMR: *δ* = 16.1, 27.9, 48.7, 83.7, 110.7, 114.5, 116.3, 119.6, 121.6, 122.4, 122.5, 128.1, 128.8, 135.8, 148.2, 149.6 ppm. IR (CHCl_3_): 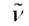 = 1725 cm^−1^. HRMS: calcd. for C_18_H_21_N_3_NaO_2_ 334.1531; found 334.1536.

*2*-[1-(3-Chloro-propyl)-1H-indazol-5-yl]-p*yrrole-1-carboxylic acid tert-butyl ester* (**5b**). Yield: 267 mg (0.74 mmol, 74%); dark orange viscous liquid; *R_f_* = 0.62. ^1^H-NMR: *δ* = 1.34 (s, 9H, CH_3_), 2.37−2.46 (m, 2H, CH_2_C*H*_2_CH_2_), 3.49 (t, 2H, CH_2_Cl, *J *= 6.3 Hz), 4.57 (t, 2H, CH_2_N, *J *= 6.3 Hz), 6.19−6.25 (m, 2H, ArH), 7.35−7.41 (m, 2H, ArH), 7.43−7.49 (m, 1H, ArH), 7.69 (m, 1H, ArH), 8.01 (s, 1H, ArH) ppm. ^13^C-NMR: *δ* = 27.9, 32.7, 42.1, 45.6, 83.7, 108.0, 110.8, 114.7, 121.3, 122.5, 123.8, 127.5, 129.1, 133.9, 135.3, 139.3, 149.6 ppm. IR (CHCl_3_): 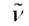 = 1725 cm^−1^. HRMS: calcd. for C_19_H_22_ClN_3_NaO_2_ 382.1298; found 382.1299.

*2-(1H-Indazol-5-yl)-pyrrole-1-carboxylic acid tert-butyl ester* (**5c**). Yield: 141 mg (0.50 mmol, 50%); dark orange viscous liquid; *R_f_* = 0.28 (hexane/ethyl acetate, 7:3). ^1^H-NMR: *δ* = 1.34 (s, 9H, CH_3_), 6.21−6.27 (m, 2H, ArH), 7.37−7.49 (m, 3H, ArH), 7.74 (s, 1H, ArH), 8.11 (s, 1H, ArH) ppm. ^13^C-NMR: *δ* = 27.9, 83.8, 109.0, 110.8, 114.8, 121.0, 122.5, 123.0, 127.7, 129.5, 135.0, 135.4, 139.6, 149.6 ppm. IR (CHCl_3_): 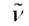 = 1734, 3469 cm^−1^. HRMS: calcd. for C_16_H_17_N_3_NaO_2_ 306,1218; found 306.1223.

*5-(1-tert-Butoxycarbonyl-1H-pyrrol-2-yl)-indazole-1-carboxylic acid tert-butyl ester* (**5d**). Yield: 310 mg (0.81 mmol, 81%); dark orange viscous liquid; *R*_f_ = 0.67 (hexane/ethyl acetate, 7:3). ^1^H-NMR: *δ* =1.35 (s, 9H, CH_3_), 1.73 (s, 9H, CH_3_), 6.26−6.21 (m, 2H, ArH), 7.38−7.36 (m, 1H, ArH), 7.52 (dd, 1H, ArH, *J* = 1.6 Hz, *J* = 8.7 Hz), 7.70−7.69 (m, 1H, ArH), 8.14 (dd, 1H, ArH, *J* = 0.7 Hz, *J* = 8.7 Hz), 8.17 (s, 1H, ArH) ppm. ^13^C-NMR: *δ* = 27.9, 28.4, 83.8, 85.2, 108.2, 111.3, 118.4, 119.0, 123.6, 124.5, 128.7, 130.3, 135.2, 135.4, 139.3, 149.3, 149.7 ppm. IR (CHCl_3_): 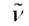 = 1736, 1737 cm^−1^. HRMS: calcd. for C_21_H_25_N_3_NaO_4_ 406.1743; found 406.1740.

*2-(1-Phenylacetyl-1H-indazol-5-yl)-pyrrole-1-carboxylic acid tert-butyl ester* (**5e**). Yield: 180 mg (0.45 mmol, 45%); yellow viscous liquid; *R*_f_ = 0.42. ^1^H-NMR: *δ* = 1.35 (s, 9H, CH_3_), 4.54 (s, 2H, CH_2_), 6.21−6.27 (m, 2H, ArH), 7.26−7.44 (m, 6H, ArH), 7.52 (dd, 1H, ArH, *J* = 1.6 Hz, *J* = 8.7 Hz), 7.71 (m, 1H, ArH), 8.17 (s, 1H, ArH), 8.41 (dd, 1H, ArH, *J* = 0.7 Hz, *J* = 8.7 Hz) ppm. ^13^C-NMR: *δ* = 27.9, 41.6, 83.8, 108.9, 112.2, 116.2, 118.3, 119.3, 123.9, 124.5, 127.4, 128.3, 129.0, 129.7, 133.4, 135.9, 138.2, 140.8, 149.7, 171.6 ppm. IR (CHCl_3_): 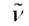 = 1731, 1739 cm^−1^. HRMS: calcd. for C_24_H_23_N_3_NaO_3_ 424.1637; found 413.1632.

*2-(1-Acetyl-1H-indazol-5-yl)-pyrrole-1-carboxylic acid tert-butyl ester* (**5f**). Yield: 97.5 mg (0.30 mmol, 30%); yellow viscous liquid; *R*_f_ = 0.79 (hexane/ethyl acetate, 9:1). ^1^H-NMR: *δ* = 1.35 (s, 9H, CH_3_), 2.83 (s, 3H, CH_3_), 6.22−6.26 (m, 2H, ArH), 7.36−7.38 (m, 1H, ArH), 7.55 (dd, 1H, ArH, *J* = 1.6 Hz, *J* = 8.7 Hz), 7.70−7.71 (m, 1H, ArH), 8.13 (s, 1H, ArH), 8.41 (dd, 1H, ArH, *J* = 0.7 Hz, *J* = 8.7 Hz) ppm. ^13^C-NMR: *δ* = 23.2, 27.9, 84.0, 110.9, 114.7, 115.3, 120.9, 122.9, 126.2, 128.7, 131.3, 131.7, 134.4, 140.8, 149.7, 171.3 ppm. IR (CHCl_3_): 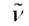 = 1719, 1778 cm^−1^. HRMS: calcd. for C_18_H_19_N_3_NaO_3_ 348.1324; found 348.1320.

*1-Ethyl-5-thiophen-2-yl-1H-indazole *(**7a**). Yield: 137 mg (0.60 mmol, 60%); brown solid, m.p. 104–106 °C; *R*_f_ = 0.55 (hexane/ethyl acetate, 6:4). ^1^H-NMR: *δ* = 1.59 (t, 3H, CH_3_, *J* = 7.3 Hz), 4.43 (q, 2H, CH_2_, *J* = 7.3 Hz), 7.07−7.10 (m, 1H, ArH), 7.25−7.30 (m, 2H, ArH), 7.39 (d, 1H, ArH, *J=* 8.7 Hz), 7.65 (dd, 1H, ArH, *J* = 1.6 Hz, *J* = 8.7 Hz), 7.94 (m, 1H, ArH), 8.01 (s, 1H, ArH) ppm. ^13^C-NMR: *δ* = 15.2, 44.1, 109.7, 118.3, 121.1, 124.4, 124.8, 125.7, 127.6, 128.2, 133.4, 138.5, 145.1 ppm. IR (CHCl_3_): 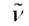 = 2988, 3002 cm^−1^. HRMS: calcd. for C_13_H_13_N_2_S 229.0799; found 229.0803.

*2-Ethyl-5-thiophen-2-yl-2H-indazole* (**7g**). Yield: 198 mg (0.87 mmol, 87%); brown solid, m.p. 100–103 °C; *R*_f_ = 0.42. ^1^H-NMR: *δ* = 1.63 (t, 3H, CH_3_, *J* = 7.3 Hz), 4.46 (q, 2H, CH_2_, *J* = 7.3 Hz), 7.06−7.09 (m, 1H, ArH), 7.25 (m, 1H, ArH), 7.30 (m, 1H, ArH), 7.58 (dd, 1H, ArH, *J* = 1.6 Hz, *J* = 8.7 Hz), 7.73 (m, 1H, ArH), 7.86 (m, 1H, ArH), 7.91 (s, 1H, ArH) ppm. ^13^C-NMR: *δ* = 16.2, 48.8; 109.7, 116.8, 118.1, 121.5, 122.5, 122.6, 122.9, 124.3, 125.7, 128.2, 145.5 ppm IR (CHCl_3_): 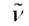 = 2978, 3037 cm^−1^. HRMS: calcd. for C_13_H_13_N_2_S 229.0799; found 229.0803.

*1-(3-Chloro-propyl)-5-thiophen-2-yl-1H-indazole* (**7b**). Yield: 171 mg (0.62 mmol, 62%); brown solid, m.p. 105–107 °C; *R*_f_ = 0.75 (hexane/ethyl acetate, 7:3). ^1^H-NMR: *δ* = 2.30−2.46 (m, 2H, CH_2_CH_2_CH_2_), 3.49 (t, 2H, CH_2_Cl, *J* = 6.3 Hz), 4.57 (t, 2H, CH_2_N, *J* = 6.3 Hz), 7.09−7.10 (m, 1H, ArH), 7.25−7.30 (m, 2H, ArH), 7.49 (d, 1H, ArH, *J* = 8.7 Hz), 7.68 (dd, 1H, ArH, *J* = 1.6 Hz, *J* = 8.7 Hz), 7.94 (m, 1H, ArH), 8.03 (s, 1H, ArH) ppm. ^13^C-NMR: δ = 32.7, 42.1, 45.6, 109.1, 118.3, 122.9, 124.5, 124.6, 125.9, 127.9, 128.2, 134.1, 139.6, 144.9 ppm IR (CHCl_3_): 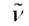 = 2966, 3001 cm^−1^. HRMS: calcd. for C_14_H_14_ClN_2_S 277.0566; found 277.0567.

*5-Thiophen-2-yl-indazole-1-carboxylic acid tert-butyl ester *(**7d**). Yield: 210 mg (0.70 mmol, 70%); brown solid, m.p. 112–113 °C; *R*_f_ = 0.39 (hexane/ethyl acetate, 9:1). ^1^H-NMR: *δ* = 1.73 (s, 9H, CH_3_), 7.11 (m, 1H, ArH), 7.30 (m, 1H, ArH), 7.33 (dd, 1H, ArH, *J *= 1.6 Hz, *J* = 8.7 Hz), 7.79 (dd, 1H, ArH, *J* = 0.7 Hz, *J* = 8.7 Hz), 7.92 (m, 1H, ArH), 8.17 (m, 2H, ArH) ppm. ^13^C-NMR: *δ* = 28.4, 85.2, 115.2, 118.0, 123.6, 125.2, 126.7, 127.8, 128.4, 130.7, 139.3, 139.8, 144.0, 149.3 ppm. IR (CHCl_3_): 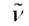 = 1743, 3001 cm^−1^. HRMS: calcd. for C_16_H_16_N_2_NaO_2_S 323.0830; found 323.0827.

*2-Phenyl-1-(5-thiophen-2-yl-indazol-1-yl)-ethanone* (**7e**). Yield: 95 mg (0.30 mmol, 30%); brown solid, m.p. 114–116 °C; *R*_f_ = 0.49. ^1^H-NMR: *δ* = 4.54 (s, 2H, CH_2_), 7.11 (m, 2H, ArH), 7.29−7.45 (m, 6H, ArH), 7.81 (dd, 1H, ArH, *J* = 1.6 Hz, *J* = 8.7 Hz), 7.93 (m, 1H, ArH), 8.17 (s, 1H, ArH), 8.43 (dd, 1H, ArH, *J* = 0.7 Hz, *J* = 8.7 Hz) ppm. ^13^C-NMR: *δ* = 41.8, 116.2, 117.8, 123.8, 125.3, 127.3, 128.3, 128.4, 128.5, 128.6, 128.8, 130.2, 130.8, 131.6, 135.2, 140.3, 171.6 ppm. IR (CHCl_3_): 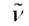 = 1713, 3001 cm^−1^. HRMS: calcd. for C_19_H_14_N_2_NaOS 341.0724; found 341.0727.

*1-(5-Thiophen-2-yl-indazol-1-yl)-ethanone *(**7f**). Yield: 85 mg (0.35 mmol, 35%); brown solid, m.p. 116–117 °C; *R*_f_ = 0.37 (hexane/ethyl acetate, 9:1). ^1^H-NMR: *δ* = 2.79 (s, 3H, CH_3_), 7.09−7.12 (m, 1H, ArH), 7.30−7.36 (m, 2H, ArH), 7.81 (dd, 1H, ArH, *J* = 1.7 Hz, *J* = 8.7 Hz), 7.92−7.94 (m, 1H, ArH), 8.13 (s, 1H, ArH), 8.43 (d, 1H, ArH, *J* = 8.7 Hz) ppm. ^13^C-NMR: *δ* = 23.2, 116.1, 117.8, 123.8, 125.3, 127.2, 128.3, 128.4, 131.5, 138.5, 139.9, 143.8, 171.5 ppm. IR (CHCl_3_): 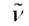 = 1713 cm^−1^. HRMS: calcd. for C_13_H_10_N_2_NaOS 265.0411; found 265.0412.

*5-(1-tert-Butoxycarbonyl-1H-pyrrol-2-yl)-1H-indazole-3-carboxylic acid methyl ester* (**10**). Compound **10** was prepared from 5-bromo-1*H*-indazole-3-carboxylic acid methyl ester **8** (61 mg, 0.24 mmol) and 2-pyrroleboronic acid **4** (99 mg, 0.47 mmol) according to the general procedure for the Suzuki coupling reaction. Yield: 57 mg (0.17 mmol, 70%); orange viscous liquid; *R_f_* = 0.27 (hexane/ethyl acetate, 1:1). ^1^H-NMR: *δ* = 1.34 (s, 9H, CH_3_), 4.05 (s, 3H, OCH_3_), 6.26−6.29 (m, 2H, ArH), 7.37−7.41 (m, 1H, ArH), 7.48 (dd, 1H, ArH, *J *= 1.5 Hz, *J* = 8.8 Hz), 7.67 (d, 1H, ArH, *J* = 8.8 Hz), 8.17−8.20 (m, 1H, ArH), ppm. ^13^C-NMR: *δ* = 27.9, 51.3, 83.8, 108.8, 110.7, 114.6, 115.0, 122.6, 123.0, 123.6, 129.1, 134.9, 140.5, 141.7, 150.2, 171.0 ppm. IR (CHCl_3_): 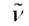 = 1728, 1774 cm^−1^. HRMS: calcd. for C_18_H_19_N_3_NaO_4_ 364.1273; found 364.1270.

*5-(1-tert-Butoxycarbonyl-1H-pyrrol-2-yl)-1-methyl-1H-indazole-3-carboxylic acid methyl ester* (**11**). Compound **11** was prepared from 5-bromo-1-methyl-1*H*-indazole-3-carboxylic acid methyl ester **9** (59 mg, 0.22 mmol) and 2-pyrroleboronic acid **4** (91 mg, 0.43 mmol) according to the general procedure for the Suzuki coupling reaction. Yield: 58 mg (0.16 mmol, 75%); brown solid, m.p. 102–104 °C; *R*_f_ = 0.35 (hexane/ethyl acetate, 1:1). ^1^H-NMR: *δ* = 1.32 (s, 9H, CH_3_), 4.01 (s, 3H, OCH_3_), 4.52 (s, 3H, NCH_3_), 6.22−6.31 (m, 2H, ArH), 7.30−7.43 (m, 1H, ArH), 7.69−7.75 (m, 1H, ArH), 7.67 (d, 1H, ArH, *J *= 8.8 Hz), 8.17−8.20 (m, 1H, ArH), ppm. ^13^C-NMR: *δ* = 27.9, 42.6, 51.5, 83.8, 108.3, 110.4, 114.3, 115.0, 122.5, 123.6, 124.0, 128.9, 134.1, 139.7, 141.8, 150.1, 171.1 ppm. IR (CHCl_3_): 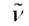 = 1716, 1774 cm^−1^. HRMS: calcd. for C_19_H_21_N_3_NaO_4_ 378.1430; found 378.1434.

## 4. Conclusions

In summary, this work establishes that indazoles bearing alkyl or acyl groups at either the *N*-1 or *N*-2 positions are suitable substrates for Suzuki cross-coupling reactions with pyrrole- and thiophene-boronic acids. We found that in the presence of Pd(dppf)Cl_2 _as palladium catalyst, the Suzuki reactions proceed in relatively short times (2 h) and in good yields. The best results were obtained when *N*-alkyl and *N*-Boc indazoles were employed as starting materials. Moreover, it was demonstrated that even bromoindazoles bearing a carbomethoxy group on *C*-3 are good coupling partners in these reactions. To the best of our knowledge, this is the first systematic study of Suzuki reactions between various 5-bromoindazoles and 2-pyrrole- or 2-thiopheneboronic acids. This could provide a promising access to new heterobiaryl compounds, valuable building blocks for use in medicinal chemistry.
